# Supplementary material for: Host transcriptional signatures associated with disease tolerance and environmental persistence in a mosquito–microsporidian system
Source: Commun Biol. 2026 Jul 4;9:1412. doi: 10.1038/s42003-026-10633-x (PMC13714254; doi:10.1038/s42003-026-10633-x)
Supplement: Supplementary file 2 [file 42003_2026_10633_MOESM1_ESM.pdf]

nature portfolio

Peer Review File

Host transcriptional signatures associated with disease tolerance and environmental persistence in a mosquito–microsporidian system

Corresponding Author: Dr Luís Silva

This file contains all reviewer reports in order by version, followed by all author rebuttals in order by version.

Version 0:

Reviewer comments:

Reviewer #1

(Remarks to the Author)
This manuscript describes a well-performed experiment and data analysis. The background and introduction are strong, and the authors demonstrate a clear understanding of disease tolerance from an ecological and evolutionary perspective. The experimental design is solid, with clear justifications, and the data is analyzed in a standard manner.

However, the manuscript is currently too descriptive. There is no experimental evidence to associate the observed transcriptional changes with disease tolerance or environmental persistence, other than correlations with existing literature. This is not enough to prove the manuscript's main conclusions. The results serve as solid preliminary data for a deeper mechanistic understanding of disease tolerance, but more work is needed.

Reviewer #2

(Remarks to the Author)
Dear Author,

I enjoyed reading your manuscript and find this study interesting. All my comments are included in the attached document (annotated manuscripts_RP), where I have commented on the manuscript using track changes (commentsRP1 to RP64).

However, here are my three main concerns regarding this study:

1. The study is largely descriptive and based on a single point in time (day 10). While the trends you report are intriguing, the conclusions should be framed in a way that reflects both the correlational nature of the data and the limitations of a single snapshot. Some interpretations, such as in Figure 3a, may overestimate the effects you observed. I think it is essential to discuss the lineage effect (see my comments on this in the manuscript).

2. Some methodological choices, such as the use of t-tests on genes for logCPM, could benefit from a brief justification or discussion of their limitations.

3. The results highlight correlations between host gene expression and parasite life cycle characteristics, but they do not provide any major mechanistic advances. It might be useful to acknowledge this explicitly in the discussion so that readers can better assess the significance of the results.

Best regards,

Romain Pigeault

Version 1:

Reviewer comments:

Reviewer #2

(Remarks to the Author)
I am satisfied with the substantial revision effort undertaken by the author following the first round of review. The author has addressed the vast majority of my questions and suggestions. The manuscript now takes considerable care in presenting the limitations of the study while appropriately highlighting its novel contributions. Below are a few minor suggestions (the line numbers correspond to those in the document: 'revised_manuscript_marked_up').

Line 1: Since the study does not investigate the parasite transcriptome itself, can the title genuinely refer to a "transcriptomic signature of environmental persistence" (given that the persistence considered here concerns spores)?
Line 47: Please avoid overgeneralization. Although this is often the case, it does not apply to all parasites (e.g., brood parasites).
Line 48: Suggested wording: "production of asexual and sexual stages".
Line 49: Suggested wording: "This may create trade-offs".
Line 54: Cases in which parasites possess a life stage allowing environmental persistence are far from being the majority. I understand the intention to introduce this concept early in the introduction (because of its relevance to your biological model), but it does not seem broadly applicable to most parasites.
At this stage, I would perhaps avoid discussing environmental persistence too extensively. More generally, in parasites capable of long environmental persistence (and thus transmission after host death), one would expect the virulence–transmission trade-off to differ substantially from that observed in parasites for which host survival is essential for transmission. However, I assume this point is addressed later in the manuscript.
Lines 69–70: "However, comparatively little is known about how host processes influence the properties of transmission stages in parasites with environmental transmission." Unless I misunderstand, is this not actually the reverse of what is being tested in this study?
Line 220: Behind the notion of timing, there is above all the issue of parasite load. It is possible that genes overexpressed in mosquitoes infected by lineage X1 at parasite density Y would also be overexpressed in mosquitoes infected by lineage X2 once this lineage reaches the same parasite density.
Lines 319–320: Suggested wording: "Parasites employ a wide range of strategies and life cycles, many of which are determined by their ecology—which itself involves a trade-off between their performance within a host and their survival from one host to another." Indeed, trade-offs fundamentally shape the ecology of organisms.
Line 383: I believe there may be a misplaced period in the sentence.
Line 387: Perhaps mention here that this type of experiment is particularly costly, and that this initial snapshot is therefore essential to determine whether a full temporal monitoring approach would be worthwhile.
Line 402: "early" should be italicized.
Line 443: Please revise the formatting/layout.
Line 467: Would it not be preferable to include a short "limitations of the study" paragraph before the conclusion, rather than repeating twice that "present data do not establish a direct causal link"?

Sincerely,
Romain Pigeault

Open Access This Peer Review File is licensed under a Creative Commons Attribution 4.0 International License, which permits use, sharing, adaptation, distribution and reproduction in any medium or format, as long as you give appropriate credit to the original author(s) and the source, provide a link to the Creative Commons license, and indicate if changes were made.
In cases where reviewers are anonymous, credit should be given to 'Anonymous Referee' and the source.
The images or other third party material in this Peer Review File are included in the article's Creative Commons license, unless indicated otherwise in a credit line to the material. If material is not included in the article's Creative Commons license and your intended use is not permitted by statutory regulation or exceeds the permitted use, you will need to obtain permission directly from the copyright holder.
To view a copy of this license, visit https://creativecommons.org/licenses/by/4.0/

Luis M. Silva
Postdoctoral fellow
Department of Zoology
University of British Columbia
Life Sciences Institute
2350 Health Sciences Mall
Vancouver, BC Canada V6T 1Z3
E-mail: luis.silva@ubc.ca

April 15th, 2026

Subject: Reply to reviewers' commentaries, manuscript ID COMMSBIO-26-0558

Dear Reviewers,

Thank you for your comments and feedback on the manuscript. I addressed all the reviewers' questions and comments separately. Below is a point-by-point reply to the issues raised, with the reviewer's commentary in regular and my response in italic. To facilitate revision, I also included a version of the manuscript and supplementary information with the highlighted changes in yellow. I hope you will find the current document suitable for publication.

Reviewer 1

This manuscript describes a well-performed experiment and data analysis. The background and introduction are strong, and the authors demonstrate a clear understanding of disease tolerance from an ecological and evolutionary perspective. The experimental design is solid, with clear justifications, and the data is analyzed in a standard manner.

However, the manuscript is currently too descriptive. There is no experimental evidence to associate the observed transcriptional changes with disease tolerance or environmental persistence, other than correlations with existing literature. This is not enough to prove the manuscript's main conclusions. The results serve as solid preliminary data for a deeper mechanistic understanding of disease tolerance, but more work is needed.

> *I agree that the original version of the manuscript overstated the extent to which these data support mechanistic conclusions. Because the study is based on a single time point and on correlation between host gene expression and independently measured parasite life-history traits, it does not establish causal relationships between specific genes and phenotypic outcomes.*
>
> *Hence, I have revised the manuscript to clarify the scope of the inferences. Rather than interpreting individual genes or modules as mechanistic determinants of virulence or environmental persistence, the results are now framed as identifying host transcriptional states that covary with evolved parasite traits across independently evolved lineages.*
>
> *This reframing emphasizes that the analyses capture how parasite strategies are expressed through the host, revealing consistent differences in host physiological states. These states can be interpreted as alternative within-host environments that differ in damage limitation, metabolism, and tissue organization, and that are expected to influence parasite performance.*

Reviewer 2

Dear Author, I enjoyed reading your manuscript and find this study interesting. All my comments are included in the attached document (annotated manuscripts_RP), where I have commented on the manuscript using track changes (commentsRP1 to RP64). However, here are my three main concerns regarding this study:

I thank the reviewer for the careful and constructive evaluation of the manuscript, as well as for the detailed annotated comments. These have substantially improved the clarity and precision of the manuscript. The main concerns raised relate to (i) the interpretation of results from a single time point, (ii) the largely correlational nature of the analyses, (iii) the role of parasite lineage structure, and (iv) the need for clearer justification of analytical choices. These points are well taken and have been addressed throughout the revised manuscript.

Specifically, I have:

- *clarified that the analyses are based on a single time point and should be interpreted as a stage-specific snapshot of host-parasite interactions,*
- *reframed all interpretations to avoid causal language and instead emphasize host transcriptional states that covary with parasite life-history traits,*
- *explicitly addressed the role of independently evolved parasite lineages and the structure they introduce into the data,*
- *and added brief justifications for key analytical choices, including differential expression testing, PCA filtering, clustering, and WGCNA parameters.*

In addition, I have revised the manuscript throughout to improve clarity, define key terms more precisely, and address all specific comments raised in the annotated document. Because this study builds on previously published work describing these parasite lines in detail (DOIs below), I have focused on including the information necessary to interpret the present results while avoiding redundancy, and have clarified key aspects such as the definition of virulence and the life-history differences between early and late lines.

https://doi.org/10.1371/journal.ppat.1013294

https://doi.org/10.1111/eva.70136

https://doi.org/10.1098/rspb.2025.1123

1. The study is largely descriptive and based on a single point in time (day 10). While the trends you report are intriguing, the conclusions should be framed in a way that reflects both the correlational nature of the data and the limitations of a single snapshot. Some interpretations, such as in Figure 3a, may overestimate the effects you observed. I think it is essential to discuss the lineage effect (see my comments on this in the manuscript).

I agree that the use of a single time point limits the interpretation of temporal dynamics. This has now been explicitly acknowledged in the Discussion, where the results are framed as a stage-specific snapshot of host–parasite interactions. I also clarify that differences between early and late infections may reflect variation in infection dynamics, including parasite load or timing.

I further agree that the analyses are correlational and do not establish causal links between host gene expression and parasite traits. The manuscript has been revised throughout to remove causal language and to emphasize that the results identify host transcriptional states that covary with virulence and environmental persistence across independently evolved parasite lineages.

Finally, I have explicitly addressed the role of parasite lineage structure. The clustering of samples by lineage is now interpreted as reflecting biological variation among independently evolved parasite

populations, consistent with the experimental design, which relies on replication across lineages for evolutionary inference.

2. Some methodological choices, such as the use of t-tests on genes for logCPM, could benefit from a brief justification or discussion of their limitations.

> *I agree that clarification of analytical choices improves transparency. Brief justifications have now been added in the Methods for key steps, including the use of gene-wise tests on* log_2*-transformed expression values, filtering of genes for PCA, the choice of k in clustering, and parameter selection in WGCNA. These additions clarify that the analytical framework follows standard practice and that the main patterns are robust to reasonable parameter choices.*

3. The results highlight correlations between host gene expression and parasite life cycle characteristics, but they do not provide any major mechanistic advances. It might be useful to acknowledge this explicitly in the discussion so that readers can better assess the significance of the results.

> *I agree that the present study does not establish mechanistic links between specific host genes and parasite traits. This limitation is now stated explicitly in the Discussion. The manuscript has been revised to position the results as identifying structured host transcriptional states associated with parasite life-history variation, rather than as demonstrating causal mechanisms. In this context, the results provide a framework for generating testable hypotheses about how host physiological states may influence parasite performance and transmission.*

<u>Comments on the manuscript file:</u>

[RP1]: Here are my initial comments after only reading the abstract of the article: Overall, this abstract does not clearly explain the study's research question and associated predictions. What justifies this study? What is the author looking for? What is he trying to test/verify? How should early or late transmission alter the host's response? I expect all these questions to be addressed in the introduction to the article. But I think it would be useful to revisit the abstract.

[RP2]: Involved in what? In the evolution of virulence, of course, but the sentence could be clearer.

[RP3]: The concept of early and late transmission is far from obvious. Perhaps just indicate the time difference between the two.

[RP4]: But is the response different between mosquitoes infected with the early or late lineage? This is unclear at this stage.

[RP5]: idem

> *The abstract has been revised to clarify the research question, explicitly define early and late parasite lineages, and better link parasite life-history strategies to expected host responses. In particular, early and late lines are now described in terms of their selection regimes and resulting differences in virulence, parasite load, and environmental persistence. (RP1-5). See lines 15-30.*

[RP6]: The sentence conflates two distinct mechanisms of virulence. Increasing virulence through greater host exploitation reflects a quantitative change in parasite density (more parasites cause more damage), whereas investing in damaging factors represents a qualitative change in per-parasite pathogenicity (each parasite causes more harm regardless of load). These are separate evolutionary axes, not simply alternative ways of “increasing virulence.” The sentence obscures a key distinction in evolutionary disease ecology between

intensity of infection and harmfulness per unit infection. Suggestions: “Parasites can increase virulence either by increasing their within-host density (greater exploitation) or by increasing the damage caused per parasite through the production of pathogenic factors.”

[RP7]: Parasites must allocate resources between within-host replication and the production of transmission stages, whether these stages are released into the environment or taken up by a vector (e.g. asexual and sexual stage of Plasmodium in vertebrate host).

[RP8]: The term adaptation is misleading because it suggests an evolutionary process, whereas it seems to me that here the author is talking about the allocation of resources between simultaneous biological functions. So, this is not “adaptation” in the Darwinian sense, but functional optimization of intra-host growth.

> *The distinction between parasite exploitation (within-host growth) and per-parasite pathogenicity has been clarified. Terminology referring to “adaptation” has been adjusted where necessary to more accurately reflect allocation and life-history trade-offs rather than evolutionary change within the experiment (RP6-8). See lines 40-42.*

[RP9]: Why is studying the transcriptional program relevant?

[RP10]: I don't understand what co-infecting refers to. Which parasite are we talking about? Plasmodium and another parasite?

[RP11]: This sentence, as written, implicitly assumes that longer persistence is intrinsically favoured, which is not what virulence–transmission theory predicts. Natural selection does not maximise persistence per se; it maximises lifetime transmission success. Longer infections are favoured only if they increase the expected number of transmission events.

[RP12]: At this stage, I'm not sure what links we're talking about here.

[RP13]: The opposition between parasite replication/spore production within mosquitoes and the persistence of spores in the environment is not conceptually straightforward. It seems to me that these two processes are not intrinsically antagonistic: prolonged growth in the vector does not necessarily reduce the durability or infectivity of spores once released (if this is the case, it must be indicated and references added). A trade-off only arises if there is an explicit constraint on resource allocation (time, energy, or physiological resources) or if vector mortality creates a risk that forces an investment decision between producing many spores quickly and producing fewer, more robust or better-differentiated spores. Without specifying such a mechanism, the current formulation implies an automatic conflict that is not biologically justified. It would therefore be clearer to frame this as a resource or timing trade-off affecting the quantity, quality, and release timing of transmission stages, rather than as a direct opposition between within-vector replication and environmental persistence.

> *The introduction has been revised to better connect parasite life-history trade-offs with host responses and to reduce overgeneralization. The role of transcriptional data in identifying host states associated with infection processes is now more explicitly stated (RP9-13). See lines 49-58.*

[RP14]: In the paragraph as written, there is a shift in focus from well-characterized phenotypic barriers in vector-borne parasites to a more speculative discussion about host genetic programs shaping environmentally transmitted stages. Suggestion: “While host genes and molecular programmes that affect parasite replication and transmission are increasingly well characterized in vector-borne systems [refs], little is known about analogous host determinants in environmentally transmitted parasites, despite theoretical predictions that host traits and environmental survival jointly shape transmission and virulence.”

> *Clarified system description and avoided overgeneralization to vector systems. See lines 59-63.*

[RP15]: I don't understand. Just before, it says that early lines kill mosquitoes earlier than late lines. What does the author mean here when he says that late lines are more virulent?

> *Improved clarity of parasite transmission description. See lines 61-63.*

[RP16]: Idem!

> *Corrected. See lines 63-65.*

[RP17]: Please add some details

> *Clarified the ecological relevance of transmission stages. See lines 64-67.*

[RP18]: It is unclear why “vector systems” are invoked here. In the An. gambiae – V. culicis interaction, the mosquito is a host rather than a vector, so there is no vector-mediated transmission. As a result, it is not obvious that transcriptomic responses observed in this system can be generalized to other vector-borne parasites.

> *Improved clarity in the description of the host–parasite interaction. See lines 66-69.*

[RP19]: Encoded in the transcriptome is a bit metaphorical. Maybe replace it with something more precise like “reflected in” or “associated with changes in”.

> *Simplified phrasing for clarity. See lines 76-80.*

[RP20]: Is the word "measured" the most appropriate?

> *Corrected. See lines 76-79.*

[RP21]: So for the three types of lineages (early, late, and unselected), the development time of microsporidia and the time required to begin sporulation are identical?

> *Sampling is now clearly described as occurring at a common sporulating stage, ensuring comparability across infection treatments. See lines 92-94*

[RP22]: Some predictions would be welcome here.

> *Improved clarity of expected transcriptional differences. See lines 96-100.*

[RP23]: Note to self: keep in mind that the parasite load is probably, according to what is said in the introduction, different between the late and early line.

> *Clarified that differences in parasite load between early and late lines may influence host transcriptional responses. See lines 129-133.*

[RP24]: Can we really talk about an evolutionary question?

Wording adjusted to avoid overstatement of the evolutionary scope of the analysis. See lines 101-105.

[RP25]: Microsporidian infection strategies

Corrected. See lines 155-158.

[RP26]: Another important difference between the early and late lines that could strongly influence the mosquito transcriptome is parasite load!

Clarified. See lines 132-135.

[RP27]: Is the amount of spores used for exposure similar between parasite strains?

Clarified that exposure conditions were standardized across treatments. See lines 132-135.

[RP28]: What is the definition of virulence here? Mortality?

Definition of virulence clarified throughout the manuscript (host mortality). See lines 33-36, for example.

[RP29]: is a commonly used percentage; if so, please indicate this.

Justification was clarified. See lines 573-576.

[RP30]: I am not sure that we can consider an axis that explains more than 16% of the total variability in the dataset to be modest. I don't know if this will be done later in the manuscript, but it would be good to discuss this large variability observed between the different replica pools within the treatment “reference”. (Were the individuals selected to constitute the different pools selected at random?)

Interpretation of PCA variance was revised and discussed as biological heterogeneity. See lines 164-168.

[RP31]: The first x label is cropped.

Figure fixed.

[RP32]: I don't really agree with this interpretation of the results. What seems much more obvious is that the points cluster by lineage (with the exception of lineages 4 – triangle). This result is very surprising because, if I understand the M&M correctly, the lineages of the different treatments have nothing to do with each other. So, how does the author explain this? How is it that the four pools of lines 1 of the three treatments are grouped together, as are the four pools of lines 2 (early and late) and the four pools of lines 3? Is there a methodological problem? Were the different lines (1-5) processed together (all lineage 1 together, all lineage 2 together, etc.) or in batches or randomly? Dimension 1 of the PCA separates lineage 4 of the Late treatment from all the others, and then dimension 2 separates lineages 1, 2, 3, 4, and 5 (regardless of the overall treatment).

Clustering by lineage explicitly addressed and interpreted as expected biological variation among independently evolved parasite populations. See lines 200-211.

[RP33]: Here, the author is talking about technical replicas, right? (the four independent pools)

Clarified distinction between replicate pools and parasite lineages. See lines 141-143, for example.

[RP34]: The sentence stating that "both early and late infections modified the reference response, but in partially overlapping and partially distinct ways" is quite vague. It does not provide the reader with a clear sense of what is shared versus unique in the transcriptomic responses. Consider clarifying this point. Suggestion: "Pairwise differential expression analyses against the reference infection showed that both early and late infections altered the mosquito transcriptome, with a subset of genes similarly regulated in both lines and additional sets uniquely affected in either early or late infections."

Clarified overlap and differences in transcriptional responses between treatments. See lines 213-218.

[RP35]: While the classification of genes into shared versus early- or late-specific responses is informative, that measurements were taken at a single time point (day 10 post-emergence). As such, the observed differences between early and late lines might reflect a temporal shift in the mosquito response rather than genuinely distinct transcriptional programs (sampling additional time points would be necessary to distinguish whether these lineage specific patterns represent true qualitative differences or simply a shift in the timing of host gene expression).

Added explicit acknowledgement that results reflect a single time point. See lines 220-222.

[RP36]: I don't really see what this figure adds (I don't understand it).

Clarified the purpose of clustering analysis in the text. See lines 229-231.

[RP37]: It remains very descriptive. Is there no way to group genes by function? Otherwise, I don't really see what this figure adds to figure 3b and c (if we don't know the function of the genes or the metabolic pathways affected).

Functional interpretation is now provided at the module level rather than the individual gene level. See lines 296-299.

[RP38]: In SupInfo, author mentions that "a conservative power of 6 was used". But β is not a universal default parameter. The choice strongly influences the network topology. This should be justified (scale-free fit? previous work?).

Justification for WGCNA parameters added. See lines 593-596.

[RP39]: X title of the panel b is cropped.

Figure fixed.

[RP40]: More details are needed. Otherwise, you have to read the supInfo to understand what has been done and what the measures of virulence, host exploitation, fecundity, etc. mean.

Clarified definitions of virulence and other life-history traits. See lines 129-133.

[RP41]: I find it difficult to see how this part of the analysis distinguishes between correlation and causation. For example, what causal link could there be between module M9 and the persistence of spores in the environment? To what extent could the over- or under-expression of genes in the host influence the survival of spores in the environment?

Revised wording to avoid causal interpretation of module–trait relationships. See lines 285-288.

[RP42]: The functional enrichment analysis shows that modules associated with early and late infections are enriched for different GO categories. However, the interpretation that infection strategies have “partitioned the host transcriptome into immune-biased and metabolically biased programmes” seems stronger than what the data support. Indeed, GO enrichment indicates differences in overrepresented functions but does not demonstrate a functional division of the transcriptome, especially given the single time point and the potential influence of parasite load or infection dynamics. A more cautious phrasing emphasizing differential enrichment rather than transcriptomic partitioning would better reflect the evidence.

Rephrased interpretation of GO enrichment to avoid overstatement. See lines 296-299.

[RP43]: The identification of hub genes based on intramodular connectivity seems appropriate within the WGCNA framework; however, the interpretation that these genes are “associated with virulence and environmental persistence” appears too strong for me. Hub genes are defined by their correlation with the module eigengene, and the modules themselves are only correlated (indirectly) with the traits, so this analysis identifies representative genes of trait-associated modules rather than candidate genes functionally linked to those traits. A more cautious wording would prevent any excessive interpretation.

Hub gene interpretation revised to avoid causal claims. See lines 305-310.

[RP44]: The selection of the top 25 genes seems arbitrary and should be justified or presented as illustrative rather than analytical.

Clarified that hub genes are illustrative of module structure. See lines 312-316.

[RP45]: After reading the manuscript, I still don't fully understand what the author means by virulence and which of the early or late lines are the most virulent.

Clarified definition of virulence throughout the manuscript. See lines 33-36.

[RP46]: For me, this is one of the critical points of the article: because gene expression was measured at a single time point, the results represent a snapshot of host responses rather than their full temporal dynamics. Infection-induced transcriptional programmes are often highly time-dependent, and patterns observed here may strongly differ at earlier or later stages. The conclusions regarding the partitioning of immune and metabolic responses should be interpreted with appropriate caution and framed as stage-specific rather than general features of the infection process.

Expanded discussion of single time point limitation. See lines 333-336.

[RP47]: I think it is important to discuss the wide variability observed between the different replicas. The second dimension of the first principal component analysis explains 16% of the total variance in the dataset! That's a lot.

Discussed variability among replicate pools. See lines 350-360.

[RP48]: This is also a critical point in the article. It seems to me that it should be explained why the points cluster much more by lineage (1-5) than by treatment! It doesn't make sense to me.

Clarified lineage-level structure and its interpretation. See lines 337-340.

[RP49]: The statement that differences between early and late infections "reflect distinct host–parasite states rather than differences in infection age" is not fully supported, because only a single time point (day 10) was measured. These differences could instead result from temporal changes in host responses or parasite dynamics related, for example, to parasite load. Several time points would be necessary to distinguish stage-specific transcriptional effects from strategy-specific ones.

Clarified that differences may reflect infection dynamics rather than distinct programmes. See lines 233-235.

[RP50]: While the patterns are indeed consistent with life-history phenotypes, caution is needed when inferring distinct host states from a single snapshot.

Interpretation revised to remain consistent with the single time point limitation. See lines 333-336.

[RP51]: Clarify what is meant by "the opposite associations of M3 and M9." Reading this, I would expect to see a negative relationship between M3 and virulence, but a positive relationship between M3 and resistance, for example.

Clarified interpretation of module–trait relationships. See lines 285-288.

[RP52]: To truly link this study to life-history phenotypes, it would be necessary to describe the effects of early and late lines on mosquito phenotypes in greater detail, for example using a table (to avoid having to flip back and forth in the manuscript or even read several other articles to fully understand yours).

Added concise description of life-history differences between parasite lines. See lines 129-133.

[RP53]: The RNA-seq workflow is generally well described, but several analytical choices would benefit from clarification or justification. See specific comments below

Clarified methodological description. See lines 552-557.

[RP54]: Can this line be considered more virulent?

Minor methodological clarification added. See lines 552-560.

[RP55]: What does "remained longer" mean? Do they take longer to develop? Or do they develop but take longer to sporulate once mature? It would be helpful to have more information.

Clarified analytical workflow description. See lines 568-573.

[RP56]: It would be really nice to add a small table to supInfo summarizing the different effects of these lines. That would avoid having to read 5 articles to get a good overview.

Clarified definitions of traits used in analyses. See lines 585-589.

[RP57]: Is there mortality among the larvae? If so, is it different between treatments?

Minor clarification implemented. See lines 585-589.

[RP58]: What does "virulence" mean here?

Clarified definition and measurement of virulence. See lines 585-589.

[RP59]: What is the CPM threshold?

CPM threshold explicitly stated. See lines 557-560.

[RP60]: Is it logCPM +1? Or just logCPM? See line 255 The author does not specify whether a pre-counting step was added prior to logarithmic transformation (as is done, for example, in edgeR/limma). Given that the choice of pseudocounting affects variance stabilization and the influence of weakly expressed genes, this parameter should be explicitly stated.

Clarified use of pseudo-count in log_2 *transformation. See lines 557-560.*

[RP61]: PCA on the "upper 75% most variable genes" Why 75%? Is it arbitrary?

Justified PCA filtering threshold. See lines 573-576.

[RP62]: Differential expression was assessed using gene-wise t-tests on log-transformed CPM values. Because RNA-seq data are count-based and may exhibit a mean–variance relationship, most current workflows use methods such as edgeR, DESeq2, or limma-voom that explicitly model dispersion and heteroscedasticity. It would be relevant to clarify the rationale for using t-tests here and how mean–variance dependence was accounted for, as this choice may affect variance estimation and false discovery control.

Clarified rationale for statistical approach. See lines 578-583.

[RP63]: The author used $|log_2FC| > 2$ and then $|log_2FC| > 1$. This gives the impression of thresholds adjusted to the analysis rather than defined a priori. Why choose a slightly more permissive threshold?

Clarified use of different fold-change thresholds. See lines 585-589.

[RP64]: k-means with k = 6. No justification, no objective criteria.

Justified choice of k-means clustering parameter. See lines 592-596.

Thank you and kind regards,

Luís M. Silva

Luis M. Silva
Postdoctoral fellow
Department of Zoology
University of British Columbia
Life Sciences Institute
2350 Health Sciences Mall
Vancouver, BC Canada V6T 1Z3
E-mail: luis.silva@ubc.ca

May 21st, 2026

Subject: Reply to reviewers' commentaries, manuscript ID COMMSBIO-26-0558

Dear Reviewer,

Thank you for your comments and feedback on the manuscript. I addressed the reviewer's questions and comments separately. Below is a point-by-point reply to the issues raised, with the reviewer's commentary in regular and my response in italic. To facilitate revision, I also included a version of the manuscript and supplementary information with the highlighted changes in yellow. I hope you will find the current document suitable for publication.

Reviewer 2

I am satisfied with the substantial revision effort undertaken by the author following the first round of review. The author has addressed the vast majority of my questions and suggestions. The manuscript now takes considerable care in presenting the present the study's limitations while appropriately highlighting its novel contributions. Below are a few minor suggestions (the line numbers correspond to those in the document: 'revised_manuscript_marked_up').

> *We thank the reviewer for the careful evaluation of the revised manuscript and for the positive assessment of the revision effort. I also thank the reviewer for the additional, constructive suggestions below, which further improve the clarity and precision of the manuscript.*

Line 1: Since the study does not investigate the parasite transcriptome itself, can the title genuinely refer to a "transcriptomic signature of environmental persistence" (given that the persistence considered here concerns spores)?

> *I thank the reviewer for this important comment. I agree that the original title could overstate the study's scope, as the analyses focus on host transcriptional responses associated with parasite lineages differing in environmental persistence rather than on the parasite transcriptome or on transcriptional mechanisms directly determining spore persistence. To better reflect the scope and interpretation of the study, I revised the title to:*
>
> *"Host transcriptional signatures associated with disease tolerance and environmental persistence in a mosquito–microsporidian system"*

Line 47: Please avoid overgeneralization. Although this is often the case, it does not apply to all parasites (e.g., brood parasites).

I thank the reviewer for this clarification. I revised the sentence to avoid overgeneralization by specifying that this applies to many parasite systems rather than parasites broadly.

Line 48: Suggested wording: “production of asexual and sexual stages”.

Thank you, this has also been corrected.

Line 49: Suggested wording: “This may create trade-offs”.

Thank you. The sentence has been changed.

Line 54: Cases in which parasites possess a life stage allowing environmental persistence are far from being the majority. I understand the intention to introduce this concept early in the introduction (because of its relevance to your biological model), but it does not seem broadly applicable to most parasites.

At this stage, I would perhaps avoid discussing environmental persistence too extensively. More generally, in parasites capable of long environmental persistence (and thus transmission after host death), one would expect the virulence–transmission trade-off to differ substantially from that observed in parasites for which host survival is essential for transmission. However, I assume this point is addressed later in the manuscript.

I thank the reviewer for this important point. I agree that the previous wording placed too much emphasis on environmental persistence at this stage of the Introduction. I therefore revised the sentence to broaden the framing and better reflect that different transmission modes can shape infection outcomes in different ways.

Lines 69–70: “However, comparatively little is known about how host processes influence the properties of transmission stages in parasites with environmental transmission.” Unless I misunderstand, is this not actually the reverse of what is being tested in this study?

I thank the reviewer for this important clarification. I agree that the previous wording implied a stronger causal role of host processes in shaping transmission-stage properties than is directly tested here. I therefore revised the sentence to more accurately reflect that the study examines host transcriptional responses associated with parasite strategies differing in environmental transmission and persistence.

Line 220: Behind the notion of timing, there is above all the issue of parasite load. It is possible that genes overexpressed in mosquitoes infected by lineage X1 at parasite density Y would also be overexpressed in mosquitoes infected by lineage X2 once this lineage reaches the same parasite density.

Thank you for this crucial point. I agree that differences observed at a single time point may partly reflect variation in parasite load and infection dynamics across lineages, rather than entirely distinct transcriptional programmes. I therefore revised the sentence to explicitly acknowledge the potential contribution of parasite load to the observed expression differences.

Lines 319–320: Suggested wording: “Parasites employ a wide range of strategies and life cycles, many of which are determined by their ecology—which itself involves a trade-off between their performance within a host and their survival from one host to another.” Indeed, trade-offs fundamentally shape the ecology of organisms.

Thank you very much for the suggestion. The sentence has been updated.

Line 383: I believe there may be a misplaced period in the sentence.

Thank you for pointing out that typo. Corrected in the new version.

Line 387: Perhaps mention here that this type of experiment is particularly costly, and that this initial snapshot is therefore essential to determine whether a full temporal monitoring approach would be worthwhile.

I thank the reviewer for this suggestion. I added a sentence clarifying that temporal monitoring experiments are particularly resource-intensive in this system and that the present study provides an initial framework for identifying whether such approaches are likely to be informative.

Line 402: “early” should be italicized.

Thank you, it has now been corrected.

Line 443: Please revise the formatting/layout.

Thank you, the reference formatting and the unnecessary period have been changed.

Line 467: Would it not be preferable to include a short “limitations of the study” paragraph before the conclusion, rather than repeating twice that “present data do not establish a direct causal link”?

I thank the reviewer for this very helpful suggestion. I agree that consolidating the main limitations into a short, dedicated paragraph improves the flow of the Discussion and avoids repeated caveats throughout the manuscript. I therefore added a brief “limitations of the study” paragraph before the Conclusions section and simplified repeated statements regarding causality elsewhere in the text.

Thank you and kind regards,

Luís M. Silva
