# Supplementary material for: Host transcriptional signatures associated with disease tolerance and environmental persistence in a mosquito–microsporidian system
Source: Commun Biol. 2026 Jul 4;9:1412. doi: 10.1038/s42003-026-10633-x (PMC13714254; doi:10.1038/s42003-026-10633-x)
Supplement: Supplementary file 3 [file 42003_2026_10633_MOESM2_ESM.pdf]

Supplementary information

for " Host transcriptional signatures associated with disease tolerance and environmental persistence in a mosquito–microsporidian system" by Luís M. Silva.

__Supplementary files index:__

Supplementary information: Supplementary tables and figures. (*current one*)

Accompanying the manuscript:

Supplementary Data 1: Differential expression (DE) between the *reference* and the *uninfected*.

Supplementary Data 2: DE between *early* and *late*, compared to the *reference.*

Supplementary Data 3: k-means reaction norms clusters.

Supplementary Data 4: WGCNA gene-module membership.

Supplementary Data 5: WGCNA module-trait correlations.

Supplementary Data 6: Gene ontology (GO) enrichment for *early*- and *late*-biased modules.

Supplementary Data 7: Hub genes in modules 3 and 9 and their expression.

Deposited in GEO:

FASTQ files: Raw RNA-sequencing data.

Deposited in Borealis dataverse:

File S10. Virulence data (and the respective readme file).

File S11. Fecundity data (and the respective readme file).

File S12. Environmental persistence data (and the respective readme file).

File S13. R script for statistical analyses and plots.

__This document contains the following:__

Supplementary Table 1. Number of genes detected per sample.

Supplementary Table 2. Principal component analysis (PCA) of the host response to the *reference* infection compared to *uninfected* hosts.

Supplementary Table 3. Principal component analysis (PCA) of the host response to the *early* and *late* infection compared to *reference* hosts.

Supplementary Figure 1. Host RNA-sequencing and parasite gene counts.

Supplementary Figure 2. Quality control clustering and PCAs.

Supplementary Figure 3. Heatmap of the log-fold change for all transcripts across all biological replicates.

Supplementary Figure 4. Volcano plots of the host response to *early*- and *late*-infections compared to the *reference* one.

Tables:

Supplementary Table 1. Number of genes detected per sample. The number of known *Anopheles gambiae* genes per parasite lineage and biological replicate (four independent pools of ten females each) is listed below.

Treatment	Lineage	Biological replicate	Sample name	Number of known genes detected
**Uninfected**	-	1	U11	9303
	-	2	U12	9304
	-	3	U13	9146
	-	4	U14	9328
Infected with **reference parasite (unselected)**	-	1	R11	8973
	-	2	R12	8547
	-	3	R13	8600
	-	4	R14	8801
**Infected** with parasites selected for **early-transmission**	1	1	E11	9433
	1	2	E12	9043
	1	3	E13	8859
	1	4	E14	8847
	2	1	E21	9113
	2	2	E22	8557
	2	3	E23	9017
	2	4	E24	9282
	3	1	E31	9122
	3	2	E32	8984
	3	3	E33	9020
	3	4	E34	8901
	4	1	E41	8799
	4	2	E42	8982
	4	3	E43	7962
	4	4	E44	9070
	5	1	E51	8807
	5	2	E52	8951
	5	3	E53	8851
	5	4	E54	8784
**Infected** with parasites selected for **late-transmission**	1	1	L11	9171
	1	2	L12	9094
	1	3	L13	8958
	1	4	L14	9107
	2	1	L21	9083
	2	2	L22	9356
	2	3	L23	9123
	2	4	L24	8929
	3	1	L31	8922
	3	2	L32	9038
	3	3	L33	9193
	3	4	L34	9161
	4	1	L41	8716
	4	2	L42	8855
	4	3	L43	8462
	4	4	L44	7629
	5	1	L51	9386
	5	2	L52	9638
	5	3	L53	9538
	5	4	L54	9136

Supplementary Table 2. Principal component analysis (PCA) of the host response to the *reference* infection compared to *uninfected* hosts. PCA variance for the analysis shown in Figure 2a (underlined values). Shown are the eigenvalues, proportion of variance, and cumulative variance explained by the first principal components describing variation in gene expression between Anopheles gambiae infected with *reference Vavraia culicis* or not (*uninfected*). These axes were used to summarise the core transcriptional response to infection that underlies subsequent comparisons with parasites evolved for *early*- or *late*-transmission.

	Eigenvalue	Proportion of variance	Cumulative variance
**PC1**	2.50×10^{3}	0.336	0.336
**PC2**	1.20×10^{3}	0.161	0.498
**PC3**	1.03×10^{3}	0.139	0.637
**PC4**	8.25×10^{2}	0.111	0.747
**PC5**	7.37×10^{2}	0.090	0.846

Supplementary Table 3. PCA of the host response to *early* and *late* infection compared to the *reference*. PCA variance for the analysis shown in Figure 3a (underlined values). Shown are the eigenvalues, proportion of variance, and cumulative variance explained by the first principal components describing variation in gene expression between *Anopheles gambiae* infected with *early* or *late Vavraia culicis* or with the *reference* lineage.

	Eigenvalue	Proportion of variance	Cumulative variance
**PC1**	1387.51	0.186	0.186
**PC2**	835.98	0.112	0.299
**PC3**	387.21	0.052	0.351
**PC4**	371.36	0.050	0.401
**PC5**	343.87	0.046	0.447

Figures:

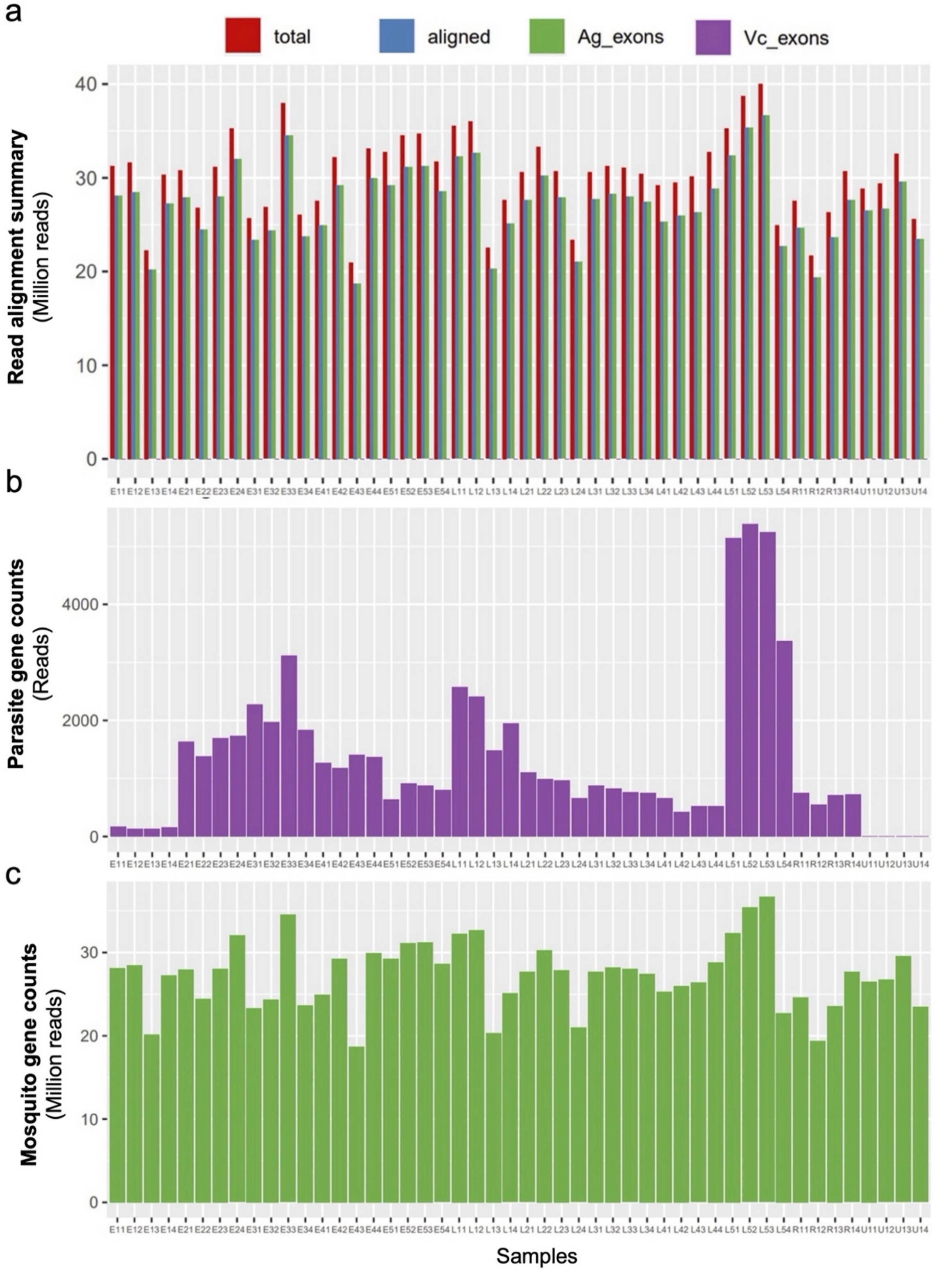

Supplementary Figure 1. Host RNA-sequencing and parasite gene counts. (a) Barplot showing the number of total reads, the number of aligned reads, and how many of which are *Anopheles gambiae* or *Vavraia culicis* exons. **(b)** Number of *V. culicis* reads found in each of the samples. **(c)** Number of *A. gambiae* reads found in each sample.

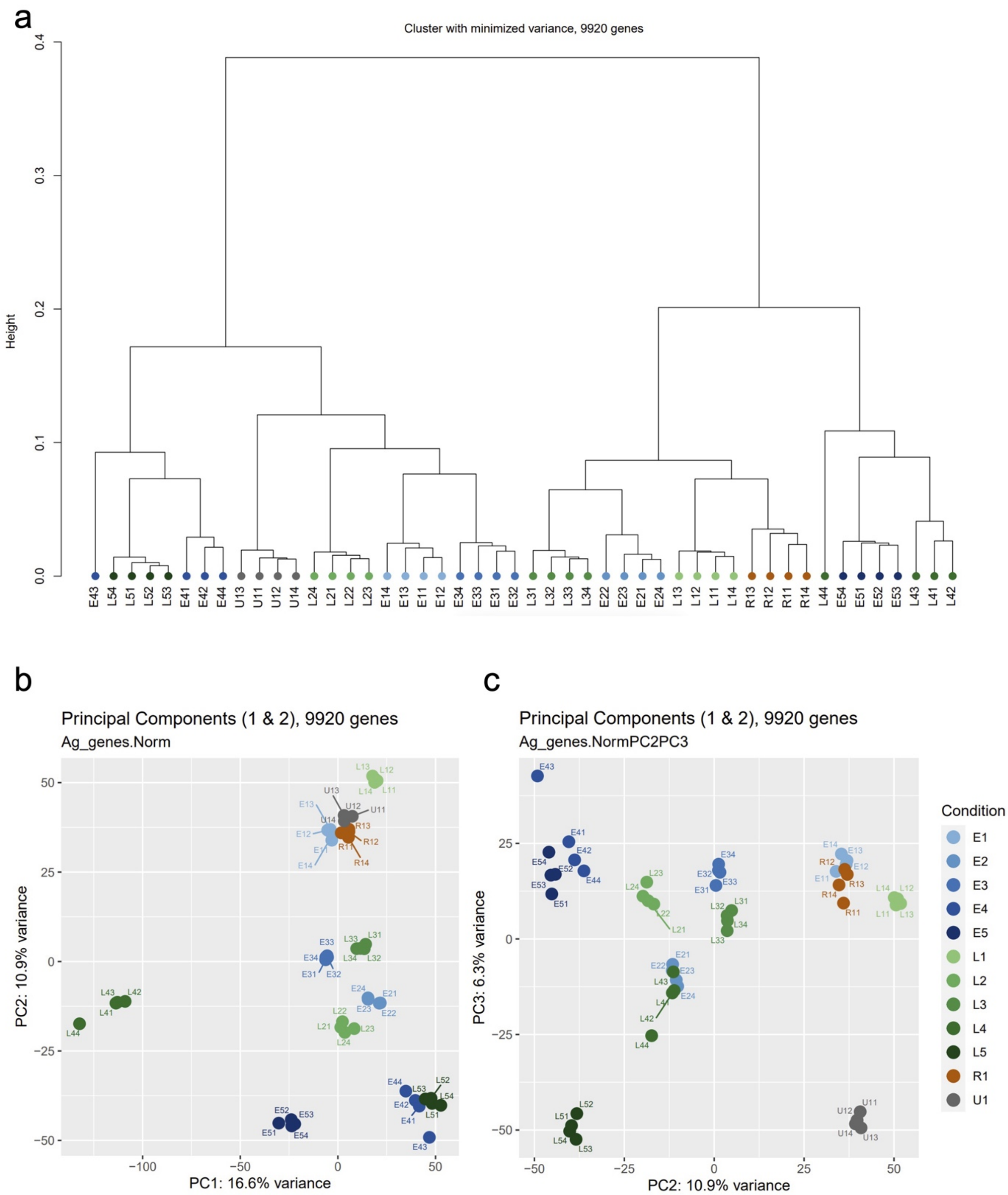

Supplementary Figure 2. Quality control clustering and PCAs. Statistical quality controls were performed through density gene expression distribution, clustering, and sample PCA. **(a)** Clustering and **(b)** PCA show that replicates group together as expected, with two exceptions for samples E43 and L44. **(c)** Since the first two principal components in the PCA explain only 30% of the variability, a PCA including PC2 and PC3 (an additional 6% of the variability) was considered. The latter does not indicate a clustering of *uninfected* and unselected *reference* infections.

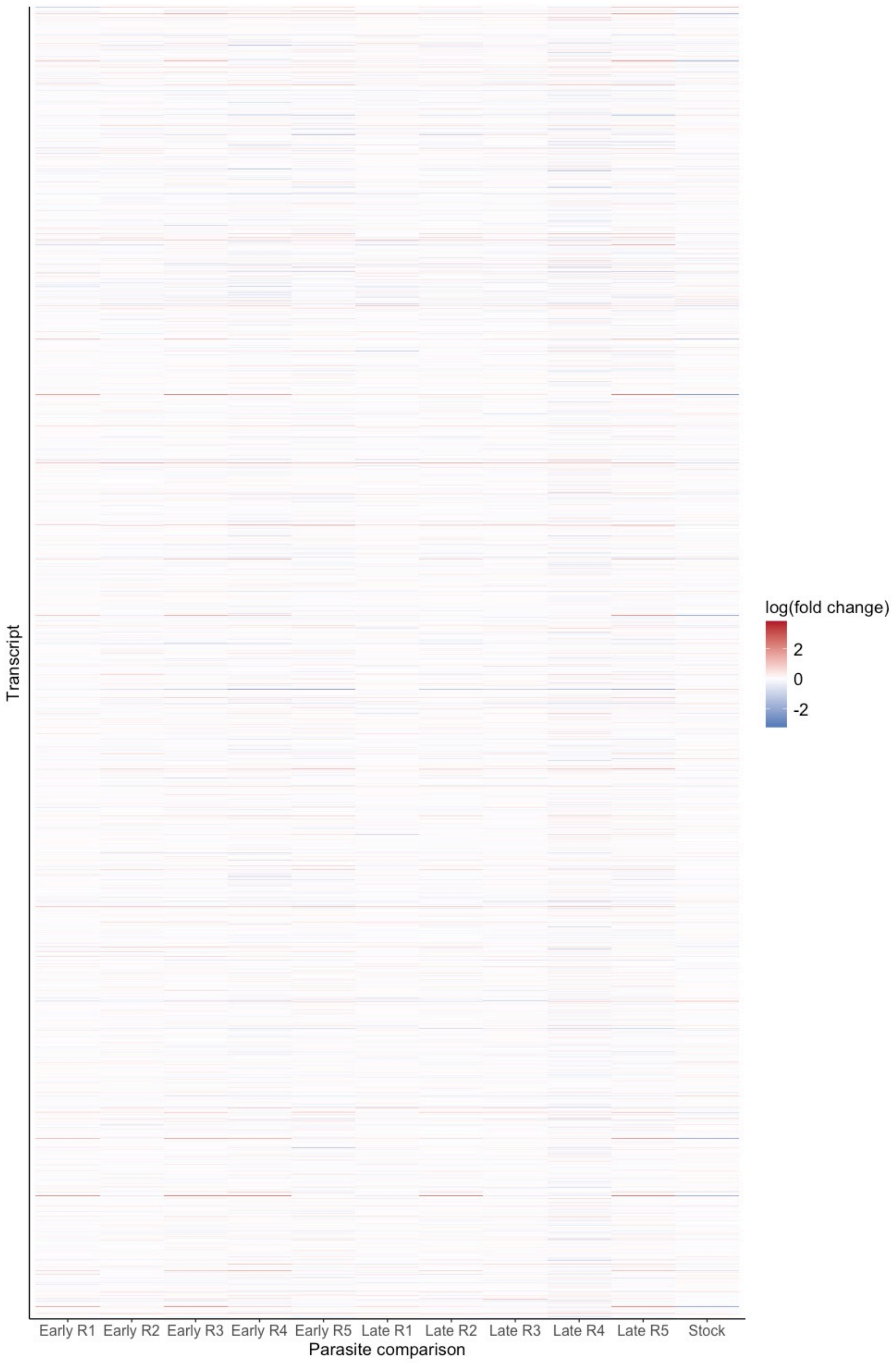

Supplementary Figure 3. Heatmap of the log-fold change for all transcripts across all biological replicates. Heatmap showing the log-transformed fold-change in the expression of all detected transcripts (*i.e.,* 9920) by parasite treatment comparison. All host samples were compared with the host samples in response to the unselected *reference/stock* parasite, except for themselves, which were compared with the *uninfected* host transcriptome.

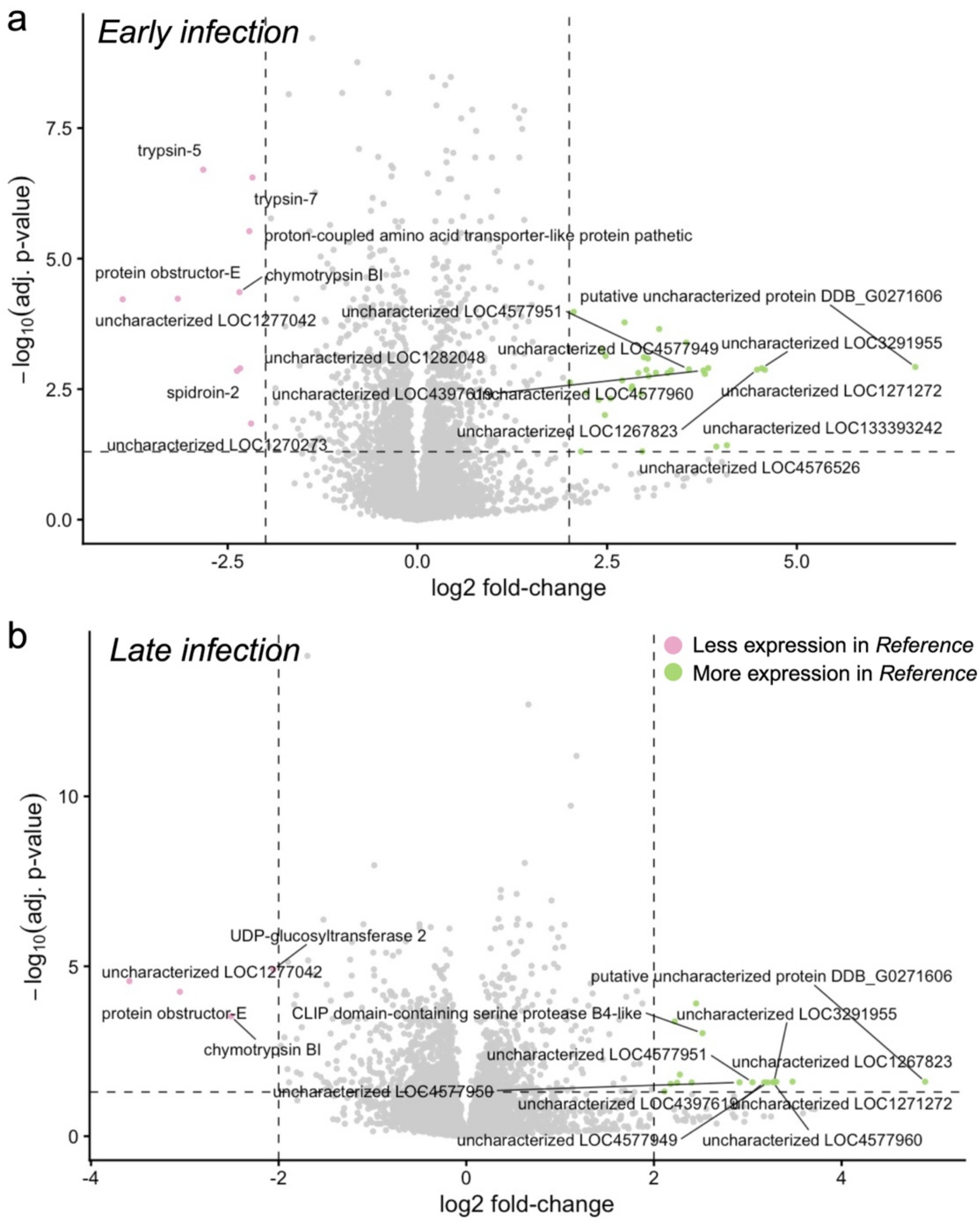

Supplementary Figure 4. Differential expression in *early*- and *late*-infections relative to the *reference* infection. (a) Volcano plot of $\log_2$ fold-change *vs.* $-\log_{10}$(adjusted *p*-value) for *early vs. reference* infections. **(b)** Volcano plot for *late vs. reference* infections. Dashed vertical lines indicate the $|\log_2$ fold-change$|$ threshold (≥ 2), and the horizontal dashed line marks the FDR cutoff (adj. $p < 0.05$). Points in pink and green denote genes with significantly lower or higher expression, respectively, in the *reference* infection compared with the evolved infection; grey points are non–differentially expressed genes. Selected labelled genes highlight those with the largest and most significant changes.
