# Supplementary material for: Host transcriptional signatures associated with disease tolerance and environmental persistence in a mosquito–microsporidian system
Source: Commun Biol. 2026 Jul 4;9:1412. doi: 10.1038/s42003-026-10633-x (PMC13714254; doi:10.1038/s42003-026-10633-x)
Supplement: Supplementary file 4 [file 42003_2026_10633_MOESM3_ESM.pdf]

Description of Additional Supplementary files

File name: Supplementary Data 1

Description: Differential expression results between Reference and Uninfected treatments.

File name: Supplementary Data 2

Description: Differential expression results between Early and Reference, and Late and Reference treatments.

File name: Supplementary Data 3

Description: k-means cluster assignments and cluster membership for all genes.

File name: Supplementary Data 4

Description: WGCNA module membership and gene level network statistics.

File name: Supplementary Data 5

Description: Module eigengene correlations with virulence, fecundity, environmental persistence, and infection treatments.

File name: Supplementary Data 6

Description: Gene Ontology enrichment analyses for differentially expressed genes and WGCNA modules.

File name: Supplementary Data 7

Description: Expression values and annotations for hub genes highlighted in the manuscript.
